# A Bibliometric Analysis of Personal Protective Equipment and COVID-19 Researches

**DOI:** 10.3389/fpubh.2022.855633

**Published:** 2022-04-29

**Authors:** Yu Zhang, Man Hu, Junwu Wang, Pingchuan Wang, Pengzhi Shi, Wenjie Zhao, Xin Liu, Qing Peng, Bo Meng, Xinmin Feng, Liang Zhang

**Affiliations:** ^1^Department of Orthopedics, Clinical Medical College of Yangzhou University, Yangzhou, China; ^2^Department of Orthopedics, Dalian Medical University, Dalian, China

**Keywords:** COVID-19, personal protective equipment, bibliometric, transmission, VOSviewer

## Abstract

COVID-19, which occurred at the end of December 2019, has evolved into a global public health threat and affects every aspect of human life. COVID-19's high infectivity and mortality prompted governments and the scientific community to respond quickly to the pandemic outbreak. The application of personal protective equipment (PPE) is of great significance in overcoming the epidemic situation. Since the discovery of severe acute respiratory coronavirus 2 (SARS-CoV-2), bibliometric analysis has been widely used in many aspects of the COVID-19 epidemic. Although there are many reported studies about PPE and COVID-19, there is no study on the bibliometric analysis of these studies. The citation can be used as an indicator of the scientific influence of an article in its field. The aim of this study was to track the research trends and latest hotspots of COVID-19 in PPE by means of bibliometrics and visualization maps.

## Introduction

The 2019 coronavirus disease (COVID-19) pandemic, which occurred at the end of December 2019, was first reported in Wuhan, Hubei Province, China (1, 2). The main clinical manifestations are fever of unknown origin, fatigue, and dry cough. Severe patients can lead to acute respiratory distress syndrome and death, accompanied by interstitial alveolar injury (3). Etiology and gene sequence analysis showed that COVID-19 was caused by severe acute respiratory coronavirus 2 (SARS-CoV-2) syndrome, which is a new member of the coronavirus family (4–6).

COVID-19 has caused serious disruption around the world. Due to the constant variation of SARS-CoV-2, people are in a state of fear and uncertainty. COVID-19 is highly contagious, with respiratory droplets and contact transmission being the main routes of transmission (7–11). For infectious diseases, controlling the source of infection, cutting off the route of transmission, and protecting the susceptible population are the three key links of infection prevention and control. For infectious diseases which are highly, it is urgent to reduce the infection rate, thus, preventing infection and blocking transmission routes are the best way to achieve this goal. In addition to the fact that vaccines can greatly reduce the morbidity, mortality, and economic losses of the disease (12, 13), personal protective equipment (PPE) can also significantly reduce the risk of exposure to infection and pollutant surfaces, and the PPE can play an important role in reducing the infection rate.

Since the discovery of SARS-CoV-2, bibliometric analysis has been widely used in many aspects of the COVID-19 epidemic, such as vaccines (12, 13), AI technology (14), and public health surveillance systems (15). Similarly, it is widely used in clinical departments, such as rheumatology (16), medical imaging (17), diabetes (18), orthopedics (19), and urology (20). Although there are many reported studies about PPE and COVID-19, there is no study on the bibliometric analysis of these studies. Citations can be used as an indicator of the scientific influence of an article in its field (21, 22). Bibliometric analysis is an important tool to help quantify the number of articles in disciplines and can provide a comprehensive overview of the literature (23, 24).

In this study, bibliometric methods were used to analyze publications on PPE and COVID-19. This study was aimed to provide a general overview of studies on PPE and COVID-19.

## Materials and Methods

### Search Methods

On 07 October 2021, the Web of Science (WOS) Core Collection database was used to identify documents on PPE and COVID-19. To ensure the breadth of the search scope, the search terms were constantly filtered. Finally, the keywords were established: TITLE = (Coronaviruses OR COVID-19 OR Coronavirus disease 2019 OR COVID-2019 OR 2019-nCoV OR nCov-2019 OR SARS-COV-2 OR Severe acute respiratory syndrome coronavirus 2 OR Novel Coronavirus) AND TITLE = (personal protective equipment OR gloves OR masks OR respirators OR goggles OR face shields OR gowns) AND Language = English AND Document type = (Article OR Review OR Letter OR Early Access OR ?Editorial Material) AND Time span = the end of December 2019 to 7 October 2021. The data were exported into Microsoft Excel 2016 and EndNote. Two duplicate articles were retrieved.

### Data Extraction and Analysis

The number of citations, authors, institutions, published journals, document types, and countries were recorded. The HistCite Pro 2.1 (http://www.histcite.com) software was used for citation analysis. Key indicators included: Local Citation Score (LCS) is the number of times this article has been referenced in the current dataset; Global Citation Score (GCS) is the number of times this article has been cited by all references in the entire WOS database; Total Local Citation Score (TLCS) is the second sum of cited frequencies of documents in the current dataset; Total Global Citation Score (TGCS) is the sum of all references cited in WOS database. The current impact factor (IF) of the journals was obtained from the Journal Citation Reports (JCR) of WOS on October 07, 2021.VOS viewer software 1.6.16 (Van Eck and Waltman, Leiden University, Leiden, Netherlands) was used for network visualization analysis (25).

## Results

The initial search resulted in 1,590 documents on PPE and COVID-19 research and a total of 1,462 documents were included in the final analysis.

A total of 1,462 documents authored by 6,993 authors and published in 750 journals were included in the final analysis. The majority of the retrieved documents consisted of articles (*n* = 778, 53.2%), followed by letters (*n* = 301, 20.6%), and editorial material (*n* = 189, 12.9%) as shown in Table 1. The most prolific author was Macintyre CR (*n* = 9, 0.13%) as described in Table 2. Among the total authors, 35 authors published at least five documents about PPE and COVID-19. Bibliometric analysis of the top 10 most contributing countries was listed in Table 3, which showed their productivity and scientific influence. Of the total countries, four countries produced more than 100 documents. The USA was the most productive country with 463 (31.7%) published documents, followed by China (*n* = 162, 11.1%), the United Kingdom (*n* = 137, 9.4%), and India (*n* = 107, 7.3%). The leading journal was Plos ONE (*n* = 34; 2.3%, IF = 3.24), followed by International Journal of Environmental Research and Public Health (*n* = 30; 2.1%, IF = 3.39), and Infection Control and Hospital Epidemiology (*n* = 26; 1.8%, IF = 3.25) as described in Table 4. The most frequently used keywords were COVID (*n* = 1,235, 38.5%), and pandemic (*n* = 576, 17.9%) as presented in Table 5. The most prolific institution was Univ Toronto (*n* = 30) as described in Table 6. Among the total institutions, 39 institutions published at least nine documents about PPE and COVID-19.

**Table 1:** Distribution of included publications by document type in COVID-19 and PPE.

**S. No**.	**Document type**	**Records**	**Percentage (%)**	**TLCS**	**TGCS**
1	Article	778	53.2	741	6,240
2	Letter	301	20.6	420	2,874
3	Editorial Material	189	12.9	324	3,039
4	Review	106	7.3	271	1,991
5	Article; Early Access	67	4.6	0	50
6	Editorial Material; Early Access	8	0.5	0	2
7	Letter; Early Access	8	0.5	0	2
8	Review; Early Access	5	0.3	0	1

*PPE, personal protective equipment; TLCS, total local citation score; TGCS, total global citation score*.

**Table 2:** Top-10 most prolific authors in COVID-19 and PPE.

**Ranking**	**Author**	**Records**	**Percentage (%)**	**TLCS**	**TGCS**
1	Macintyre CR	9	0.13	8	288
2	Szarpak L	8	0.11	12	45
3	Bialynicki BR	7	0.10	2	55
4	Chou R	7	0.10	25	60
5	Li J	7	0.10	5	32
6	Smereka J	7	0.10	12	55
7	Dana T	6	0.09	25	60
8	Filipiak KJ	6	0.09	12	45
9	Hamzavi IH	6	0.09	24	112
10	Jungbauer R	6	0.09	25	60

*PPE, personal protective equipment; TLCS, total local citation score; TGCS, total global citation score*.

**Table 3:** Top-10 most productive countries in COVID-19 and PPE.

**Ranking**	**Country**	**Records**	**Percentage (%)**	**TLCS**	**TGCS**
1	USA	463	31.7	575	5,103
2	China	162	11.1	429	2,835
3	England	137	9.4	278	2,086
4	India	107	7.3	43	349
5	Italy	89	6.1	90	705
6	Canada	87	6.0	254	1,513
7	Australia	66	4.5	51	675
8	Germany	47	3.2	78	357
9	Japan	47	3.2	15	226
10	Spain	46	3.1	17	286

*PPE, personal protective equipment; TLCS, total local citation score; TGCS, total global citation score*.

**Table 4:** Top-10 leading journals in COVID-19 and PPE.

**Ranking**	**Journals**	**Records**	**Percentage (%)**	**TLCS**	**TGCS**	**IF (2020)**	**Quartile**
1	Plos ONE	34	2.3	0	258	3.24	1
2	International Journal of Environmental Research and Public Health	30	2.1	0	207	3.39	2
3	Infection Control and Hospital Epidemiology	26	1.8	10	185	3.25	2
4	BMJ-British Medical Journal	21	1.4	0	425	39.89	1
5	Journal of Hospital Infection	21	1.4	63	298	3.93	1
6	Annals of Internal Medicine	18	1.2	87	286	25.39	1
7	American Journal of Infection Control	17	1.2	18	90	2.92	1
8	Science of The Total Environment	16	1.1	0	420	7.96	1
9	Journal of The European Academy of Dermatology and Venereology	15	1.0	31	113	6.17	1
10	Scientific Reports	12	0.8	0	34	4.38	1

*PPE, personal protective equipment; TLCS, total local citation score; TGCS, total global citation score; IF impact factor*.

**Table 5:** Top-10 frequently used words in COVID-19 and PPE.

**Ranking**	**Word**	**Records**	**Percentage (%)**	**TLCS**	**TGCS**
1	COVID	1,235	38.5	1,351	10,836
2	Pandemic	576	17.9	583	4,924
3	Mask	464	14.5	443	3,446
4	Masks	423	13.2	736	4,859
5	Protective	420	13.1	394	4,731
6	Personal	400	12.5	379	4,672
7	Face	397	12.4	687	5,078
8	Equipment	394	12.3	369	4,629
9	Use	226	7.0	378	2,500
10	SARS	160	5.0	582	3,782

*PPE, personal protective equipment; TLCS, total local citation score; TGCS, total global citation score*.

**Table 6:** Top-10 most prolific institutions in COVID-19 and PPE.

**Ranking**	**Author**	**Records**	**TLCS**	**TGCS**
1	Univ Toronto	30	26	224
2	Harvard Med Sch	27	17	255
3	Univ Hong Kong	22	251	1,098
4	Univ Milan	17	23	154
5	Oregon Hlth and Sci Univ	16	32	766
6	Univ Penn	16	37	246
7	Wroclaw Med Univ	16	20	136
8	All India Inst Med Sci	15	1	31
9	Stanford Univ	15	1	146
10	Johns Hopkins Univ	14	0	143

*PPE, personal protective equipment; TLCS, total local citation score; TGCS, total global citation score*.

### Network Visualization Map of Co-authorship Country

**Figure 1:**
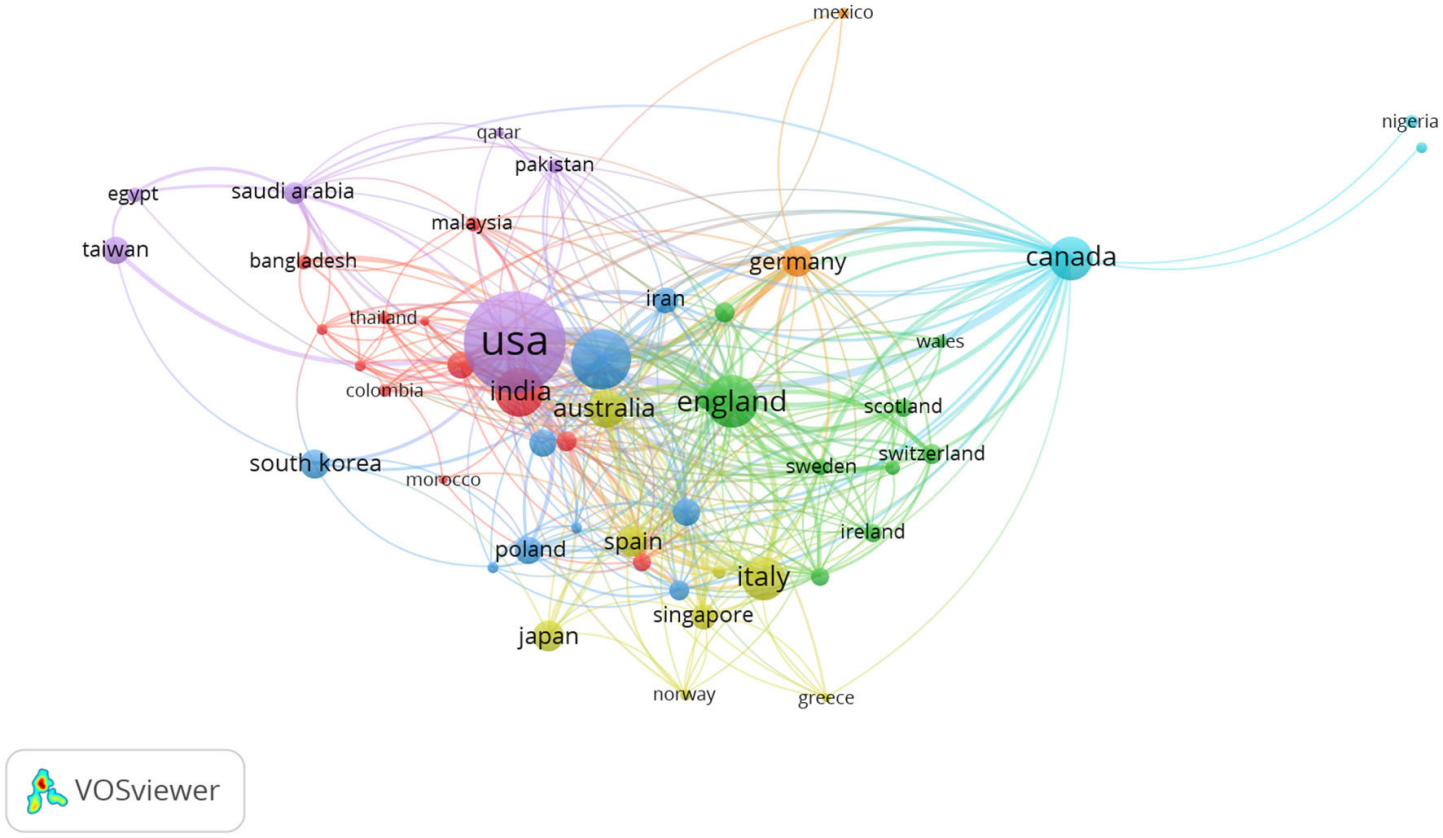
Network visualization map of co-authorship country. A minimum of five documents per country was fixed. Of the 94 countries, only 55 meet the threshold. The size of the circle represents the number of articles published by the country, and the larger the circle, the higher the country's contribution to co-authorship. The more connections between the two countries, the stronger cooperation exists between the two countries.

Considering masses of countries, a minimum of five documents per country was fixed. Of the 94 countries, 55 countries satisfied this condition. Table 3 describes a complete picture of the academic performance of leading countries. The size of the circle represents the number of articles published by the country, and the larger the circle, the higher the country's contribution to co-authorship. The thicker the lines between the two countries, the closer the cooperation exists between the two countries (Figure 1). The USA was the most productive country, with 463 published documents and total link strength (TLS) of 219, making it a country with the largest network of international cooperation. China ranked second in the number of published documents and third in TLS. The strongest country linkages were between the USA and Canada (*n* = 21).

### Network Visualization Map of Keyword Analysis

**Figure 2:**
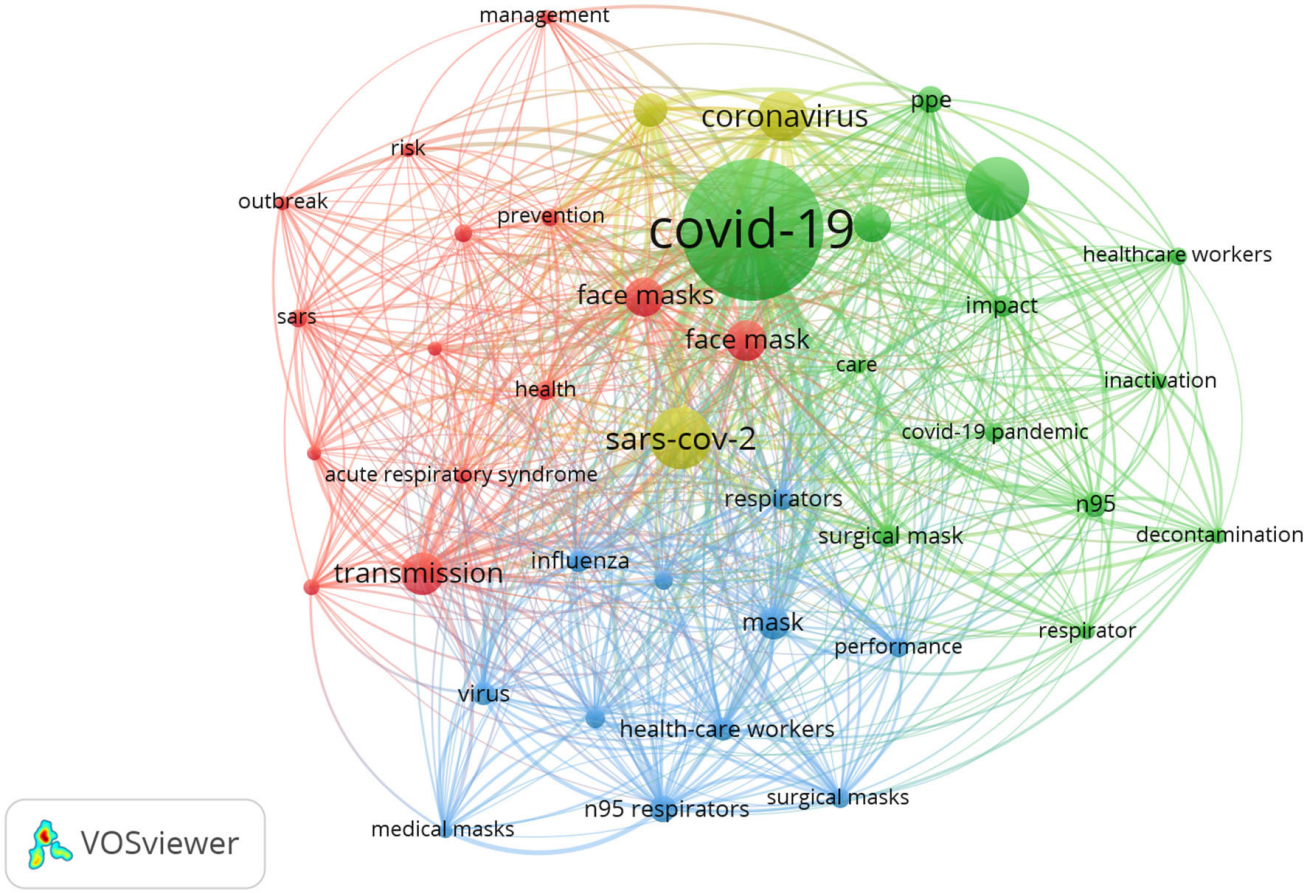
Network visualization map of keyword analysis. The co-occurrence network analysis tool was used to set the minimum number of occurrences to 18. Of the 3,061 keywords, 41 met the threshold. The larger the circle was, the words were used more frequently. Forty-one keywords classified in major four clusters.

The keyword analysis is one of the most important indicators of bibliometrics. According to co-occurrence analysis, the relationship of items is based on the number of publications in which they occur together (26). The co-occurrence network analysis tool was used to set the minimum number of occurrences to 10. Of the 3,061 keywords, 75 met the threshold. The keyword “COVID-19” (total link strength 1,382) appeared most, with 597 co-occurrences, followed by SARS-CoV-2 (occurrences = 165, TLS = 554, 11.7%), and PPE (occurrences = 175, TLS = 464, 9.8%; Figure 2).

### Network Visualization Map of Active Journals

**Figure 3:**
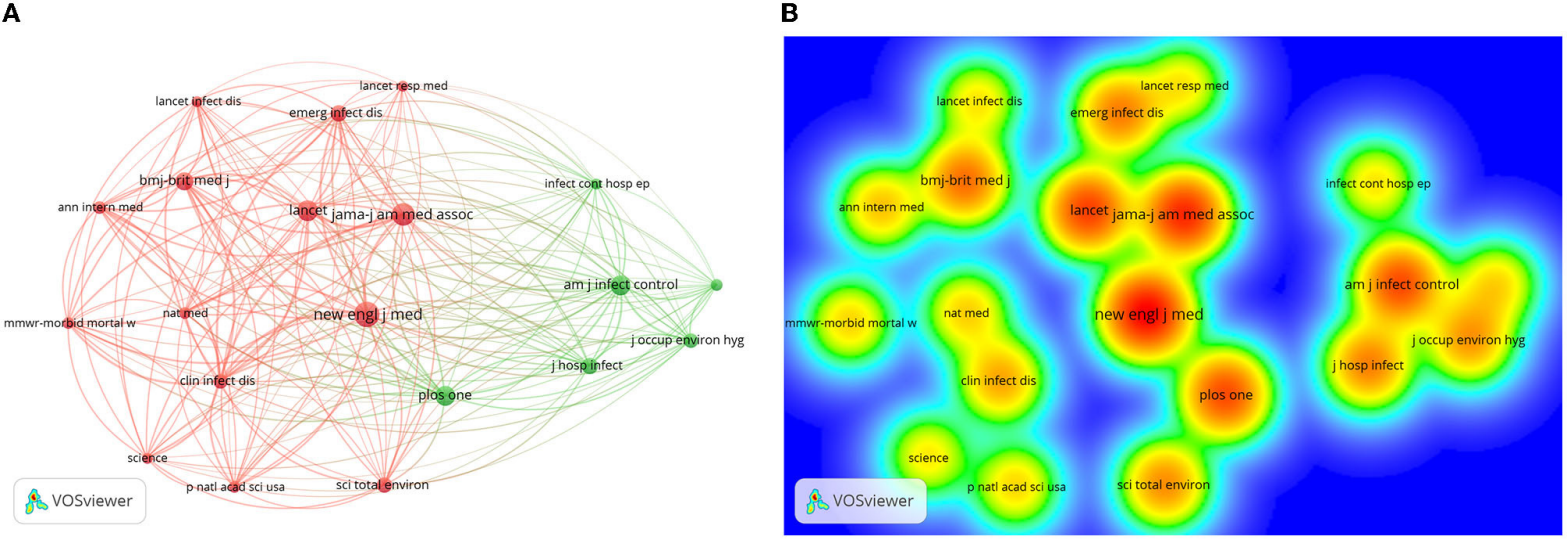
Visualization mapping of co-citation cited sources. (**A)** Network visualization map; **(B)** density visualization map. A minimum number of citations of a source: 200. Of the 11,711 sources, 20 sources met the threshold. For each of the 20 sources, the TLS with other sources was calculated. The sources with the greatest TLS were selected.

The minimum number of citations of a source was set at 200. Of the 11,711 sources, only 20 sources met the threshold. The New England Journal of Medicine was the leading source with the highest TLS 22,151 (citations = 775), followed by JAMA—Journal of the American Medical Association (TLS = 19,601, citations = 650), and Plos One (TLS = 17,525, citations = 543). The strongest link (1,070) was between the New England Journal of Medicine and the JAMA—Journal of the American Medical Association (Figure 3A). Two clusters of sources were identified by this analysis. Cluster 1, red color, included 14 journals closely in terms of scope. The New England Journal of Medicine was at the core of this cluster. Cluster 2, green color, including six sources and the American Journal of Infection Control was in the core. The areas of the red color in Figure 3B indicated active sources that have the highest rate of co-citation (i.e., New England Journal of Medicine).

### Network Visualization Map of Co-authorship Institutions

**Figure 4:**
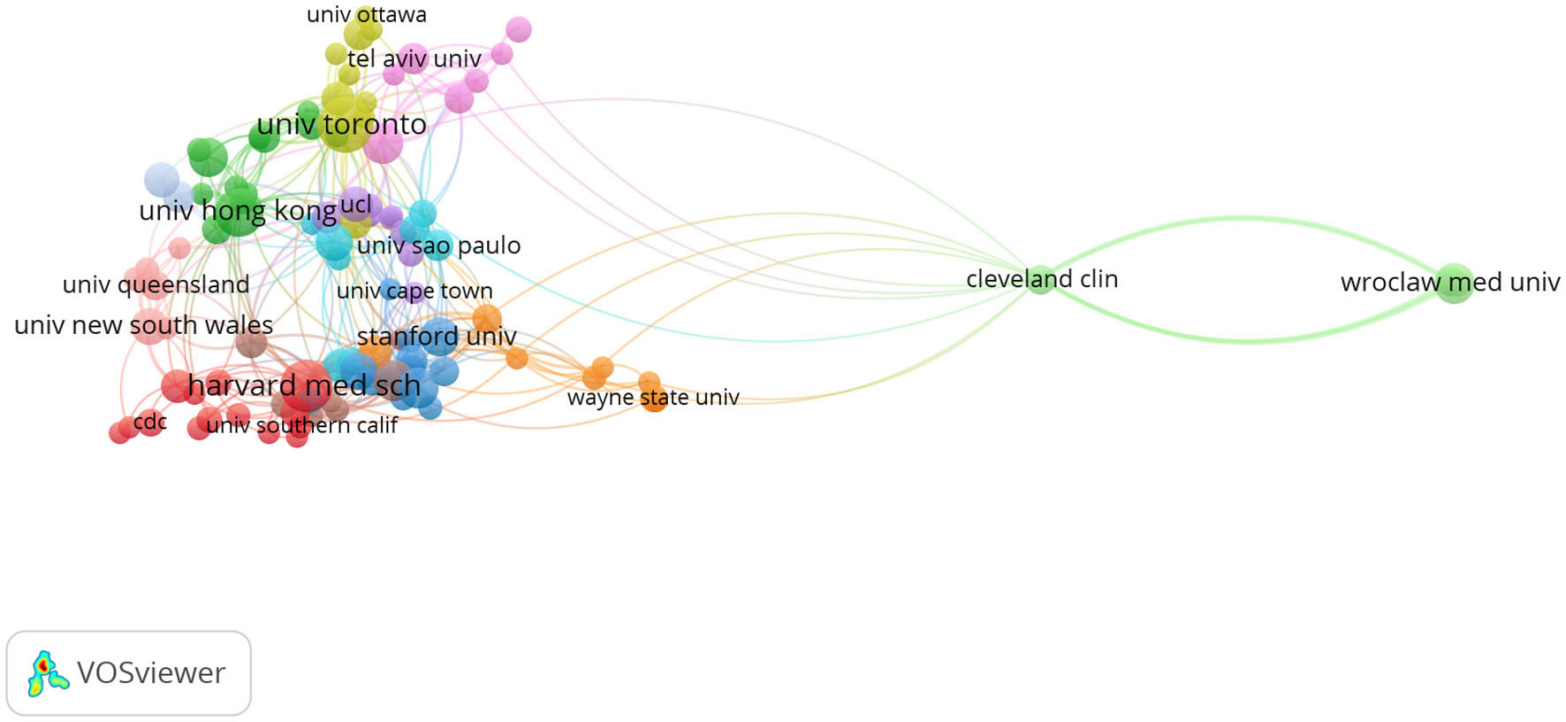
Network visualization map of co-authorship institutions. A minimum of five documents per organization was fixed. Of the 2,403 institutions, 116 meet the threshold.

In the visualization map, 116 institutes published more than five articles, and the cooperation network of institutions was shown in Figure 4. There were 388 links of collaboration with a TLS of 585. The University of Toronto had the highest number of links and the highest TLS (25 links with a TLS of 39).

## Discussion

The coronavirus disease, which occurred at the end of December 2019, has evolved into a global public health threat (27) and affects every aspect of human life. COVID-19's high infectivity and mortality prompted governments and the scientific community to respond quickly to the pandemic outbreak. The previous literature on COVID-19 was mainly devoted to the use of vaccines (12, 13) and therapeutic drugs (28, 29). However, as the virus is still mutating, it is particularly important to block the route of transmission to prevent the further spread of the epidemic. The purpose of this study was to track the research trends and latest hotspots of COVID-19 in PPE by means of bibliometrics and visualization maps. Bibliometrics analysis is a form of statistical analysis of published articles (23, 24). Based on these technologies, we can analyze various aspects, such as countries, institutions, sources, authors, and journals. These technologies are widely used in different scientific fields, from micro (institutional level) to macro (global level), which can be evaluated qualitatively and quantitatively. The present study was the first bibliometric study to focus on COVID-19 and PPE research and visualization mapping. The results of this study were helpful to collate the data and easily obtain the maximum yield data of COVID-19 and PPE, as well as the current research, focuses on COVID-19 in PPE and other major bibliometrics information.

In the present study, a total of 1,462 documents on COVID-19 and PPE were analyzed. The most frequent keyword and author keywords' co-occurrence was COVID-19, which was in line with other studies (12, 14). It showed that the research on COVID-19 was still a hot topic in academic circles.

Sharing very early information with countries, research institutes, government organizations, researchers, and the general public play a key role in the early stage of outbreaks and epidemics (30–32). According to this information, we can take various protective measures. In the early days of the COVID-19 outbreak, China began to share existing information about SARS-CoV-2 with other countries to study a variety of PPE, treatments, and vaccines.

The most prolific authors in COVID-19 and PPE research were from Australia (Macintyre CR). By analyzing the main authors in this field, we can identify the main contributors and look for opportunities for further cooperation. Among the types of documents, besides Articles, Letters, and Editorial Material attracted more attention. Most people thought that this was one of the most informative documents in the early days (12).

The impact factor of the top-10 journal ranged from 2.92 “American Journal of Infection Control” to 39.89 “BMJ-British Medical Journal”, of which eight journals were placed in Quartile 1 (Q1) and 2 in Quartile 2 (Q2). This finding showed that the authors targeted top journals. The current analysis indicated that most of the publications on COVID-19 were published in influential and well-known journals. Many journals, which have special issues on COVID-19 have always been considered a priority and published in an open-access model (33). The USA was the highest productive country. According to the early bibliometric analysis, China was the main country of COVID-19, and the reason may be that this disease has first appeared in China. A few months later, there were a large number of COVID-19 cases in the United States, and the publishing trend and COVID-19 trend transferred to the United States. The difference in the volume of contributions in each country can be attributed to the following factors: the wealth of the country, development level, population size, scientific capacity, and scientific infrastructure. Another major factor was related to the prevalence of COVID-19 in different countries. All these factors were related to the prevalence of epidemics, which was a major factor that cannot be ignored. This forced countries with high prevalence to strive to combat the impact of COVID-19's spread, and this analysis revealed the leading role played by the United States and China, which was due to COVID-19's high prevalence in these countries (34, 35).

In the visualization map of the institutions, the University of Toronto had the highest number of links and highest TLS. It was based on the two-dimensional space of cooperative relations between institutions. This cooperation was conducive to producing high-impact scientific research on the basis of complementary practice, experience, and skills (36). In this epidemic, cooperation between research centers around the world has a great advantage in fighting the epidemic (13, 37).

### Limitations

Although bibliometrics is an effective method to evaluate article influence, there are still several limitations in our current research. First, only WOS was used to search the literature, not the existing Google academic, Medline, or other databases (38). The number of citations in the report may be slightly different. Second, English was included in the choice of language, which may lead to the omission of related articles in other languages (39). Third, the number of citations may be higher for the older research, but the older articles may not keep up with current research hotspots (40, 41). Finally, one of the reasons for a high number of citations may be self-citation, including authors citing their own articles and authors citing more articles from the journals they want to publish in (42). Further research is needed to analyze the frequency of self-citation and its influence on the article. Despite these limitations, bibliometric analysis is still an important tool to help quantify the number of articles in disciplines and provide a comprehensive overview of the literature. Our study is the first bibliometric analysis of COVID-19 and PPE research and visualization mapping. Moreover, our analysis can track the research trends and latest hotspots of COVID-19 in PPE.

## Conclusion

This is the first bibliometric study to focus on COVID-19 and PPE research and visualization mapping, and this study provides detailed information on published literature and overall research perspective. The United States is the most productive country, and the University of Toronto is the most active institution. The most frequent keyword and author keywords' co-occurrence are COVID-19. The result is helpful for the funding agencies to evaluate the research trends of global COVID-19 and PPE. The application of PPE, by blocking the route of transmission, greatly reduced the prevalence of COVID-19, not only to protect themselves but also conducive to the health of others. The use of PPE is still a hot zone of future research.

## Data Availability Statement

The original contributions presented in the study are included in the article, further inquiries can be directed to the corresponding author.

## Ethics Statement

Our study was a retrospective assessment of the public data, so the approval of the institutional review committee is not required.

## Author Contributions

YZ and MH: protocol/project development, data analysis, and manuscript writing. JW, PW, PS, BM, and XF: data collection or management and data analysis. WZ, XL, and QP: assist in the literature searching based on WOS and data analysis. LZ: protocol/project development, data analysis, and manuscript editing. All authors are agreed and approved the final manuscript for publication.

## Funding

This study was funded by the Project on Maternal and Child Health Talents of Jiangsu Province (F201801).

## Conflict of Interest

The authors declare that the research was conducted in the absence of any commercial or financial relationships that could be construed as a potential conflict of interest.

## Publisher's Note

All claims expressed in this article are solely those of the authors and do not necessarily represent those of their affiliated organizations, or those of the publisher, the editors and the reviewers. Any product that may be evaluated in this article, or claim that may be made by its manufacturer, is not guaranteed or endorsed by the publisher.

## Abbreviations

PPE: personal protective equipment
COVID-19: 2019 coronavirus disease
WOS: Web of Science
LCS: Local Citation Score
GCS Global Citation Score
TLCS: Total Local Citation Score
TGCS: Total Global Citation Score
IF: impact factor
JCR: Journal Citation Reports.
